# Fecal microbiota transplantation mitigates bone loss by improving gut microbiome composition and gut barrier function in aged rats

**DOI:** 10.7717/peerj.12293

**Published:** 2021-10-21

**Authors:** Sicong Ma, Ning Wang, Pu Zhang, Wen Wu, Lingjie Fu

**Affiliations:** Shanghai Key Laboratory of Orthopaedic Implants, Department of Orthopaedic Surgery, Shanghai Ninth People’s Hospital, Shanghai Jiao Tong University School of Medicine, Shanghai, China

**Keywords:** Fecal microbiota transplantation, Osteoporosis, Intestinal barrier, 16S rRNA gene sequencing, Aged rats

## Abstract

**Background:**

Gut microbiota (GM) dysbiosis is closely related to bone loss and the occurrence of osteoporosis in animals and human. However, little is known about the effect and the mechanisms of fecal microbiota transplantation (FMT) on bone in the treatment of senile osteoporosis.

**Methods:**

Aged female rats were randomly divided into the FMT group and the control group. 3-month-old female rats were used as fecal donors. The rats were sacrificed at 12 and 24 weeks following transplantation and the serum, intestine, bone, and feces were collected for subsequent analyses.

**Results:**

The bone turnover markers of osteocalcin, procollagen type 1 N-terminal propeptide (P1NP), and carboxy-terminal peptide (CTX) decreased significantly at 12 and 24 weeks following FMT (*P* < 0.05). At 12 weeks following transplantation, histomorphometric parameters including the bone volume (BV), trabecular bone volume fraction (BV/TV), trabecular number (Tb.N), and trabecular thickness (Tb.Th) of the FMT group were comparable to the control group. However, at 24 weeks following transplantation, these parameters of the FMT group were significantly higher than those of the control group (*P* < 0.05). Besides, the GM aggregated at 12 and 24 weeks following FMT, and the ecological distance was close between the rats in the FMT group and the donor rats. Alpha diversity, shown by the Shannon index and Simpson index, and the Firmicutes/Bacteroidetes ratio decreased significantly after FMT at 24 weeks. Furthermore, FMT restored the GM composition in aged rats at the phylum and family level, and the intestinal microbiota of the aged rats was similar to that of the donor rats. Correlation network analysis indirectly suggested the causality of FMT on alleviating osteoporosis. FMT improved the intestinal structure and up-regulated the expression of tight junction proteins of occludin, claudin, and ZO-1, which might be associated with the protective effects of FMT on bone.

**Conclusions:**

GM transplanted from young rats alleviated bone loss in aged rats with senile osteoporosis by improving gut microbiome composition and intestinal barrier function. These data might provide a scientific basis for future clinical treatment of osteoporosis through FMT.

## Introduction

Feces are common medicinal materials in traditional Chinese medicine. In the past, fresh or fermented fecal suspensions which were called yellow soup had been used to treat digestive diseases including abdominal pain, diarrhea, and constipation (Zhang et al., 2012). At present, fecal microbiota transplantation (FMT) has become an effective medication strategy to treat a variety of metabolic diseases.

Osteoporosis is characterized by low bone mass and skeletal fragility, which results in increased susceptibility to low-trauma fractures (Black & Rosen, 2016). The consequences of osteoporotic fractures including re-fractures, high rates of mortality and morbidity, and economic costs remain a huge social problem in older populations. Primary osteoporosis can be divided into senile osteoporosis and postmenopausal osteoporosis, which are induced by aging and estrogen deficiency, respectively.

The gut microbiota (GM) is defined as microorganisms colonized in the digestive tract, which plays a crucial role in a variety of diseases such as type 2 diabetes, fatty liver, and intestinal disorders (Li et al., 2020; Lang & Schnabl, 2020; Kappler et al., 2020; Soleimanpour et al., 2020). Firmicutes and Bacteroidetes are the major phyla of GM in feces followed by Proteobacteria and Actinobacteria (Locantore et al., 2020). Disorders of GM have been linked with several metabolic diseases such as Alzheimer’s disease, sarcopenia, stroke, cardiovascular disease, and osteoporosis (Chen et al., 2020; Picca et al., 2019; Battaglini et al., 2020; Liu et al., 2020; Behera et al., 2020).

Our recent studies using ovariectomized and aged rats have demonstrated the altered GM composition and intestinal function in osteoporosis (Ma et al., 2020a, 2020b). GM disorder is also related to osteoporosis in postmenopausal women (He et al., 2020). Furthermore, Das et al. (2019) analyzed the changes of GM in patients with osteopenia or osteoporosis and those with normal bone mineral density (BMD) (*n* = 181). Results showed that the changes of intestinal microbiota and bone mass were the most correlated (Das et al., 2019). Another study in elderly Chinese people showed that *Roseburia*, *Bifidobacterium*, and *Lactobacillus* were positively correlated with BMD, and larger amounts of lipopolysaccharides biosynthesis were synthesized in low-BMD individuals (*n* = 102) (Li et al., 2019). Moreover, significant differences in bacterial taxa and diversity were found among normal individuals and those with osteoporosis, or osteopenia (*n* = 18) (Wang et al., 2017). Therefore, the evidence strongly confirmed the intimate relationships between GM and osteoporosis and the potential of GM modulation for the treatment of osteoporosis.

FMT has the potential to treat several diseases as a novel medication (Markey, van den Brink & Peled, 2020). Recently, it has become a feasible clinical method to treat intestinal dysbiosis-related diseases (Ejtahed et al., 2016; Eiseman et al., 1958). FMT was included in the guideline for the treatment of *Clostridioides difficile* infection (CDI) (Hocquart et al., 2018). Moreover, increasing numbers of studies have shown promising results of FMT in treating inflammatory bowel disease, alcohol-induced liver injury, Alzheimer’s disease, depression, type 2 diabetes, and cerebral ischemic stroke (Groen & Nieuwdorp, 2017; Bibbò et al., 2020; Ebrahimzadeh Leylabadlo et al., 2020; Antushevich, 2020; Zhang et al., 2019; de Groot et al., 2020). It can be expected that restoration of disturbed intestinal microbiota would become a promising therapeutic method for systemic metabolic disorders.

However, little is known about the effect of FMT on bone mass in the treatment of osteoporosis. We hypothesized that FMT could alleviate bone loss in osteoporosis by restoring GM homeostasis. Therefore, this study would provide new insights into the clinical treatment of osteoporosis.

## Materials and Methods

### Experiment animals

The experimental protocol of animal trials was approved by the Ethical Committee of the Ninth People’s Hospital of Shanghai Jiao Tong University School of Medicine (SH9H-2019-A17-1). Thirty-two 18-month-old and four 3-month-old female Sprague Dawley (SD) rats were purchased from SLAC Laboratory Animal Company (SLAC., China, SCXK2012-0002) and raised in the specific pathogen-free Laboratory animal room in the Ninth People’s Hospital. The 18-month-old female SD rats were randomly divided into two groups: the FMT group (FMT, *n* = 16) and the control group (Con, *n* = 16). Rats in each group were housed as 2/cage with a 12-h light-dark cycle and free access to water and pelleted rodent diet. As the donors of fecal samples, 3-month-old female SD rats were raised in the same condition. FMT was performed by oral gavage, and rats were sacrificed by rapid cervical dislocation after 12 and 24 weeks following transplantation. The serum, intestine, bone, and feces were collected for subsequent analyses.

### Fecal microbiota transplantation

FMT was performed based on an established method (Chang et al., 2015; Di Luccia et al., 2015). Briefly, fresh fecal samples from four 3-month-old donor rats were collected under aerobic and sterile conditions by pressing the end of rectum before sacrificing (Costello et al., 2019). Every 100 mg mixed fecal samples were re-suspended in one mL of sterile saline. The suspension was vigorously vortexed for 10 s before centrifugation at 800G for 3 min. The supernatant was collected and frozen at −80 °C. Diluted supernatant was orally given to recipient rats (one mL/rat) three times per week. Normal saline was given to the control group following the same method.

### Bone mineral density detection

To verify the rat model of senile osteoporosis, bone mineral density (BMD) was measured by dual-energy X-ray absorptiometry (DXA, Hologic Discovery A, USA).

### Micro-CT analysis

Micro-CT scanning was performed using a high-resolution micro-CT 81 system (Scanco Medical AG, Bruettiselien, Switzerland) (Fu et al., 2020). Before scanning, rats were sacrificed, and tibia samples were collected and fixed in 4% paraformaldehyde. Histomorphometric parameters including bone volume (BV), trabecular bone volume fraction (BV/TV), trabecular number (Tb.N), trabecular thickness (Tb.Th), trabecular spacing (Tb.Sp), and bone surface area ratio (BS/BV) were calculated. Three-dimensional reconstruction of the tibia metaphysis was performed using the built-in Scanco software.

### ELISA analysis

The serum was homogenized and used to measure the concentration of osteocalcin (OC), P1NP and CTX using ELISA kits (XLCC, Shanghai YBL Biomedical Technologies, China).

### 16S rRNA gene sequencing and bioinformatic analysis

Fecal samples were collected from both groups and immediately stored at −80 °C for subsequent 16S rRNA gene sequencing (Ma et al., 2020a, 2020b). For the analysis of the phylogenetic GM composition, the V3–V4 region was amplified using the Illumina MiSeq instrument.

The data of 16S rRNA sequencing were analyzed as previously described (Ma et al., 2020a, 2020b). We grouped the high-quality DNA sequences into operational taxonomic units (OTUs) and compared them with the SILVA reference database (V138) at 97% similarity. The minimum sample size was deemed to be the criteria of data normalization. We performed community richness and diversity analyses (Shannon and Simpson indexes) using Mothur software (V1.44.1). To clarify the similarity or difference of data, we conducted unweighted and weighted UniFrac Principal Co-ordinates Analysis (PCoA) by calculating the ecological distance to obtain the eigenvalues and eigenvectors between samples. ThetaYC algorithm was used to calculate the similarity of the community structure of each sample at the level of OTU = 0.03. Taxonomy was assigned using the online software RDP classifier (80% threshold) based on the Ribosomal Database Project. We performed Redundancy analysis (RDA) using the vegan package in R. We used the KEGG database to predict functional pathways and define significant differences of KEGG ortholog abundances between two groups as *P* < 0.05, fold change >2 or <−2.

### Hematoxylin & eosin staining

The distal ileum was fixed in 4% (v/v) paraformaldehyde and embedded in paraffin, and then cut into four µm sections in thickness. The sections were then stained with HE. Morphological characteristics were examined under the Olympus light microscope (Japan).

### Immunohistological staining

Ileum paraffin sections were routinely dewaxed in xylene and dehydrated in gradient alcohol. After blocking with 0.3% triton X-100, in Tris-HCl buffer (0.1 M) containing 5% fetal bovine serum, the slides were incubated with the primary antibody including anti-ZO-1 (1:100, NOVUS), anti-claudin (1:100, SAB signalway antibody) and anti-occludin (1:100, Proteinbtech) at 4 °C over-night. Secondary antibodies used in this study included Cy3-or Alexa Fluor 488-conjugated donkey anti-mouse or rabbit IgG (1:400 dilution; Jackson ImmunoResearch Labs, Inc.). Nuclei were counterstained with 40, 6-diamidino-2-phenylindole (DAPI) (1:30,000 Roche Biochemicals, Monza, IT) and mounted with Vectashield (Vectorlabs, Burlingame, CA). Images were digitally captured by FV1,000 confocal laser-scanning microscope (Olympus, Japan) and processed with FV10-ASW Viewer 1.7 and Adobe Photoshop CS3. The quantitative analysis was performed by ImageJ software (NIH, Bethesda, Maryland, USA).

### Statistical analysis

All data were processed by GraphPad Prism 6.0 statistical software. The data were analyzed by Student t-test or multiple t-tests, and corrected by Holm-Sidak method, with α = 0.05. All the measurement data are expressed as mean ± standard error (SE). The statistical significance was as follows: *P < 0.05; ^#^*P* < 0.01.

## Results

### Successful establishment of the animal model of senile osteoporosis

**Figure 1:**
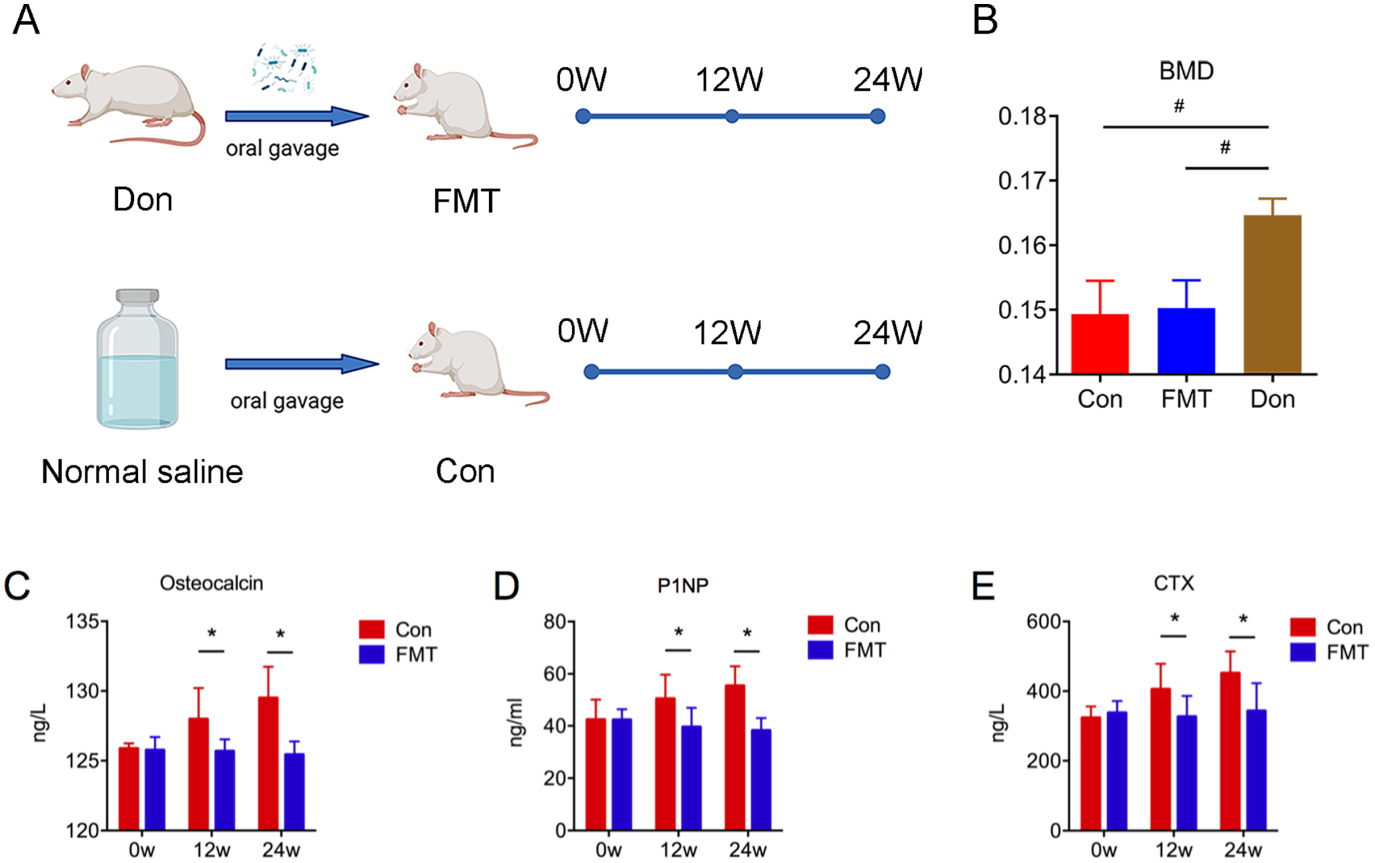
GM from young rats reduced bone turnover in aged rats of senile osteoporosis. (A) Experiment protocol. (B) Bone mineral density (BMD) comparison verified the animal model of senile osteoporosis. (C–E) Compared with control, the level of osteocalcin, P1NP and CTX in the FMT group decreased significantly at 12 and 24 weeks. *P < 0.05, ^#^*P* < 0.01. FMT: fecal microbiota transplantation; Con: control; Don: donor; N.S: normal saline.

The experiment flow of the current study was described in the study design (Fig. 1A). DXA was used to measure the BMD in order to verify the animal model of senile osteoporosis. The BMD of the FMT group (0.1503 ± 0.0018 g/cm^2^) and the control group (0.1494 ± 0.0021 g/cm^2^) was significantly lower than that of the 3-month-old rats (0.1,647 ± 0.0013 g/cm^2^, *P* < 0.01) (Fig. 1B), which demonstrated that senile osteoporosis was successfully established in the 18-month-old SD rats used in this study.

### GM from young rats reduced bone turnover in aged rats of senile osteoporosis

The bone turnover markers of OC, P1NP, and CTX increased significantly in the control group at the 12th and 24th weeks (Figs. 1C–1E), while these parameters did not change significantly in the FMT group. The OC of the FMT group decreased significantly at the 12th and 24th weeks, compared with the control group (Con12: 127.99 ± 0.81; FMT12: 125.68 ± 0.81; Con24: 129.49 ± 0.99 ng/l; FMT24: 125.43 ± 0.99 ng/l; *P* < 0.05) (Fig. 1C). Serum P1NP and CTX of the FMT group followed the same trends as OC at the 12th and 24th weeks post-transplantation (*P* < 0.05) (Figs. 1D, 1E). We concluded from the data that FMT could reverse the high bone turnover status of senile osteoporosis.

### GM from young rats alleviated bone loss in aged rats

**Figure 2:**
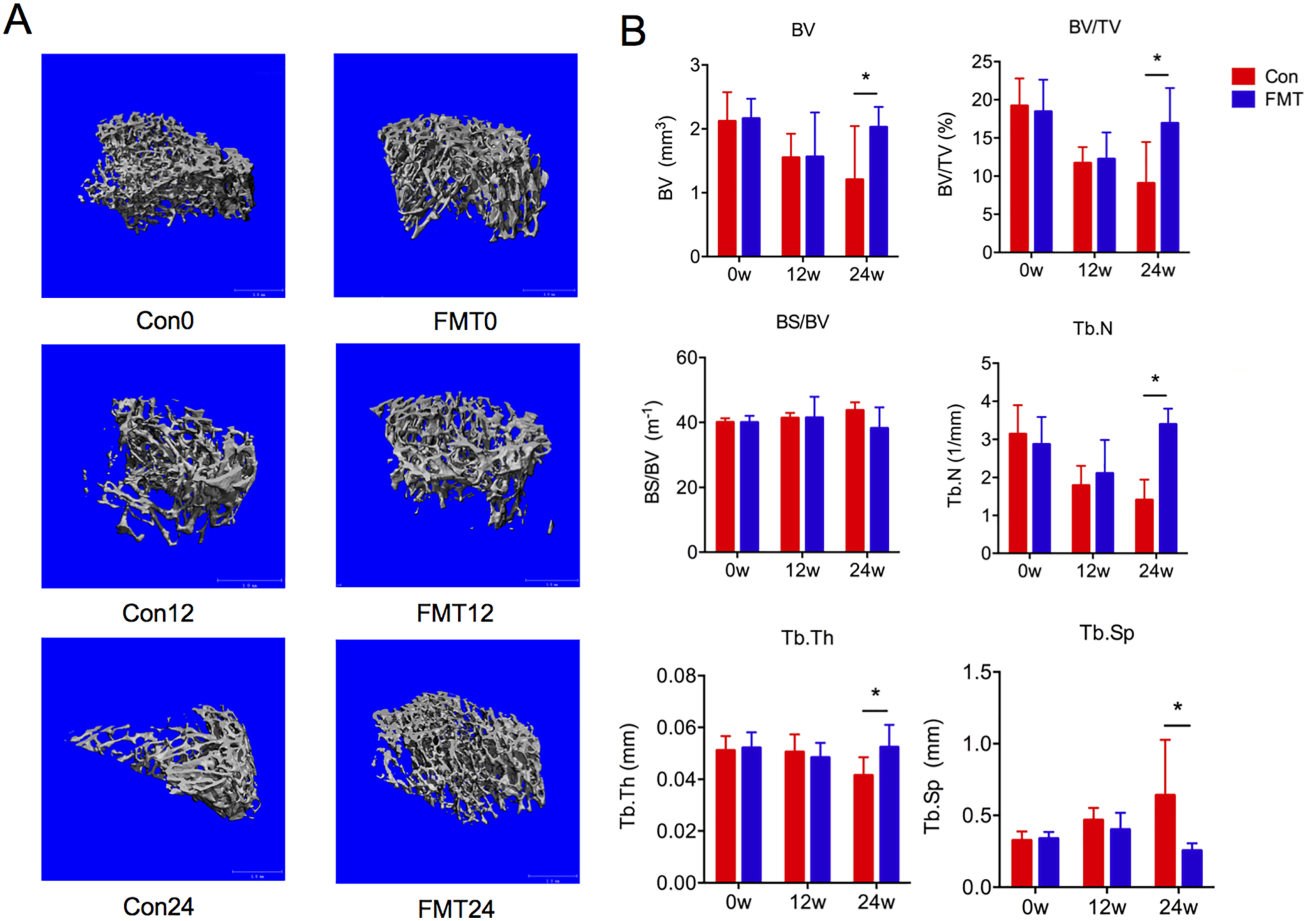
GM from young rats alleviated bone loss in aged rats. (A) Three-dimensional reconstruction of proximal tibia of the FMT group and the control group at 12 and 24 weeks. (B) Bone histomorphometric parameters comparison: bone volume (BV), bone volume density (BV/TV), specific bone surface (BS/BV), trabecular number (Tb.N), trabecular thickness (Tb.Th), and trabecular spacing (Tb.Sp). *P < 0.05. FMT: fecal microbiota transplantation; Con: control.

Three-dimensional images showed that the bone structure of the control group was gradually deteriorated during oral gavage, while little changes of the bone structure were found in the FMT group (Fig. 2A). Quantitative analyses demonstrated that BV, BV/TV, Tb.N, Tb.Th in the control group decreased gradually at the 12th and 24th weeks (Fig. 2B). At the 12th week, these parameters in the FMT group were comparable to the control group. However, at the 24th week post-transplantation, the BV (Con24: 1.20 ± 0.37; FMT24: 2.03 ± 0.37), BV/TV (Con24: 9.05 ± 2.73; FMT24: 16.93 ± 2.73), Tb.N, and Tb.Th of the FMT group were significantly higher than the control group (*P* < 0.05). These findings together with the serum bone turnover markers suggested that FMT could reverse osteoporosis in aged rats.

### GM from young rats improved the gut microbiota dysbiosis in aged rats of senile osteoporosis

**Figure 3:**
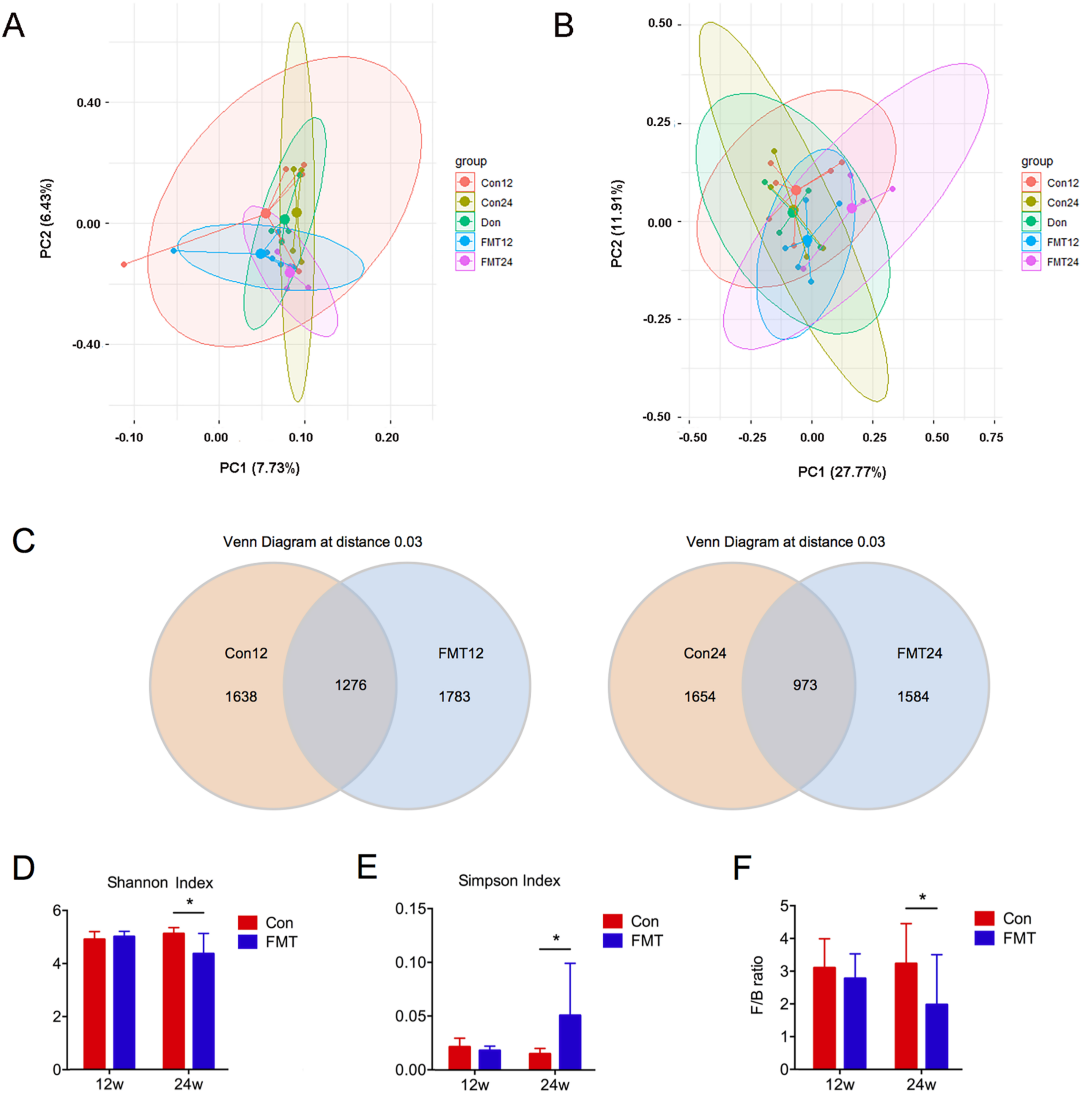
GM from young rats improved the gut microbiota dysbiosis in aged rats of senile osteoporosis. (A, B) Unweighted and weighted UniFrac principal coordinate analysis (PCoA) of all samples showed that the β-diversity of gut microbiota changed significantly after transplantation. (C) Number of common and specific OTUs of samples with OTU = 0.03. (D, E) Community diversity assessment including the Shannon index and the Simpson index showed that α diversity of gut microbiota in the FMT group decreased significantly. (F) Compared with control, the F/B value of FMT24 decreased significantly. *P < 0.05. FMT: fecal microbiota transplantation; Con: control; Don: donor.

Unweighted and weighted UniFrac PCoA was conducted to analyze the beta diversity of GM. The sample distribution of the control group was scattered, which was consistent with our previous findings in senile osteoporosis (Ma et al., 2020b) (Figs. 3A, 3B). However, the GM aggregated at 12 and 24 weeks following FMT, and the ecological distance was similar between the rats in the FMT group and the donor rats (Fig. 3A). These results demonstrated that the diversity of GM changed significantly after FMT, and the intestinal microbiota of the aged rats appeared to be similar to the donor rats.

The total number of OTUs between the two groups was significantly different at the 12th and 24th weeks post-transplantation (Fig. 3C). Shannon and Simpson indexes are commonly used to evaluate the alpha diversity of GM. A low Shannon index and high Simpson index mean low alpha diversity. Therefore, these two indexes showed that the alpha diversity was significantly decreased at 24 weeks after FMT (*P* < 0.05) (Figs. 3D, 3E). Firmicutes/Bacteroidetes (F/B) ratio is an indicator of GM disorder. High F/B ratio was associated with disease status (Everard & Cani, 2013). The F/B ratio decreased significantly at 24 weeks after FMT, suggesting that the status of GM disorder was alleviated (*P* < 0.05) (Fig. 3F).

### GM from young rats improved GM composition in aged rats at the phylum and family levels

**Figure 4:**
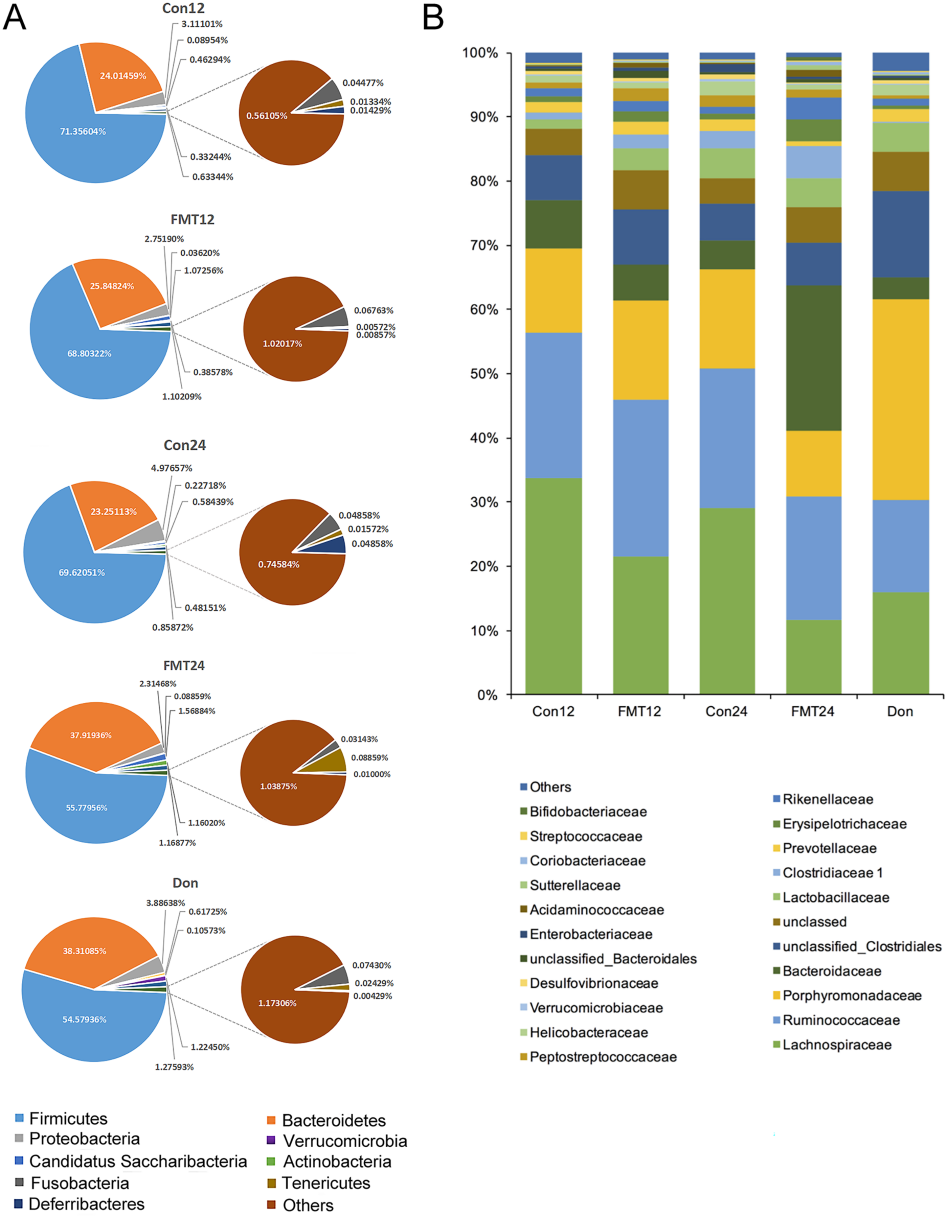
GM from young rats changed the composition of gut microbiota in aged rats at phylum and family level. (A) Pie chart of gut microbiota composition of the FMT group and the Con group at phylum level. (B) The family level composition comparison. FMT: fecal microbiota transplantation; Con: control; Don: donor.

Next, we compared the phylum-level GM contents of the two groups after transplantation (Fig. 4A). The abundance of Firmicutes of the control group was maintained at nearly 70% at the 12th and 24th weeks. However, it decreased from 68.80% at the 12th week to 55.78% at the 24th week in the FMT group, which is quite close to that of the donor rats (54.58%). The abundance of Bacteroidetes was another major phylum of gut microbiota. The abundance of Bacteroidetes in the FMT group at the 24th week (37.92%) was higher than that of the control group (23.25%), which was also similar to the donor rats (38.31%) (Fig. 4A). Furthermore, the abundance of Lachnospiraceae decreased gradually from the 12th week to the 24th week following FMT, which was also similar to that of the donor rats (Fig. 4B). These data suggested that FMT could restore GM composition in rats with osteoporosis at the phylum and family levels.

### GM from young rats changed GM composition in aged rats at the genus level

**Figure 5:**
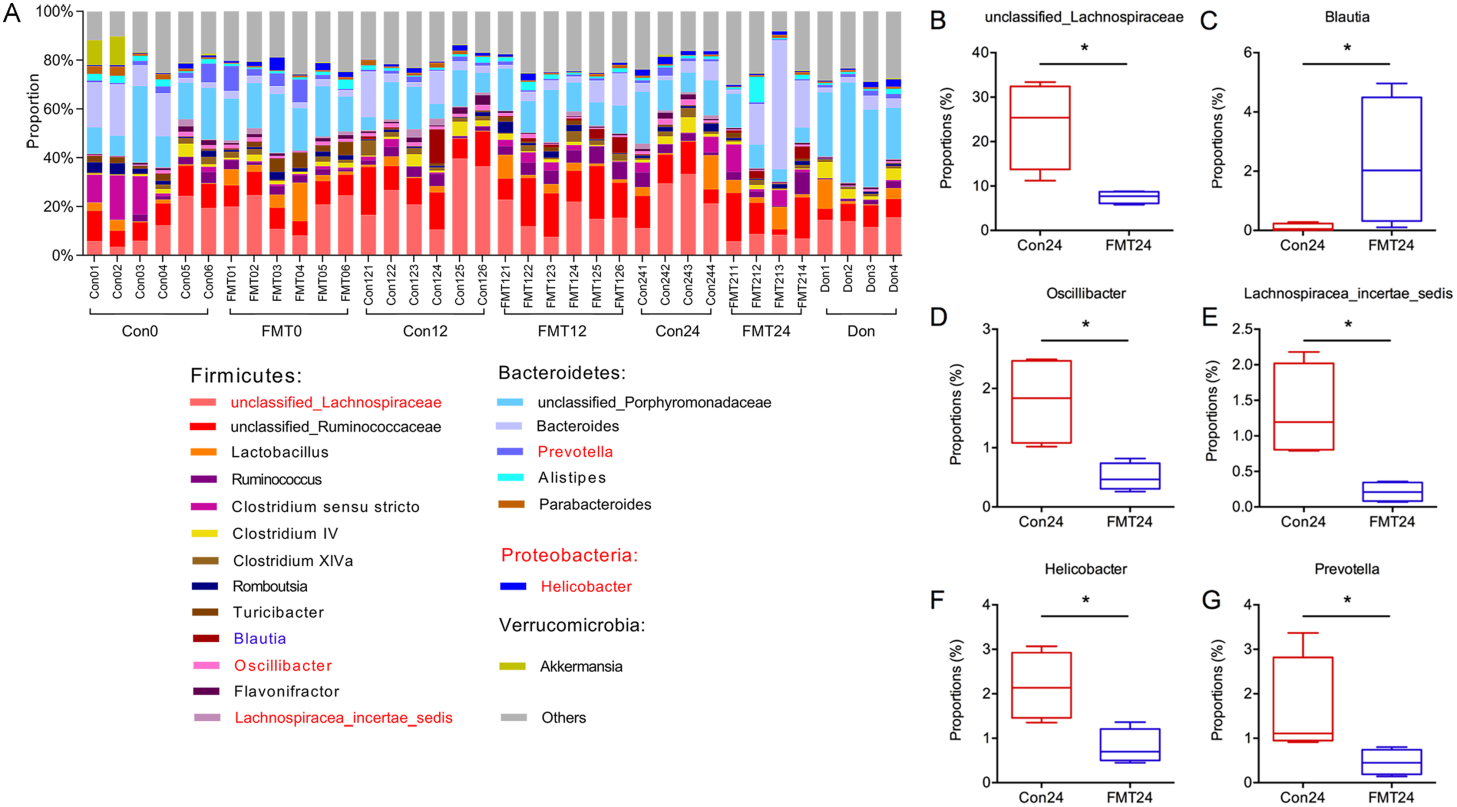
GM from young rats changed GM composition in aged rats at the genus level. (A) The relative abundance of GM at the genus level. (B–G) The unclassified_Lachnospiraceae, Blautia, Oscillibacter, Lachnospiracea_incertae_sedis, Helicobacter, and Prevotella were compared at 24 weeks. *P < 0.05. FMT: fecal microbiota transplantation; Con: control; Don: donor.

To further investigate the mechanisms of the beneficial effect of FMT on osteoporosis, we constructed a histogram of the main GM abundances of the two groups at different time points (Fig. 5A). Compared with the control group, the contents of *unclassified_Lachnospiraceae*, *Oscillibacter*, *Lachnospiraceae_incertae_sedis* decreased significantly at the 24th week (Figs. 5B, 5D, 5E) (*P* < 0.05), which was similar to the composition of the donor rats. *Blautia* increased significantly after FMT (Fig. 5C) (*P* < 0.05). Meanwhile, the content of *Helicobacter* and *Prevotella* decreased significantly following FMT (Figs. 5F, 5G).

### RDA analysis of GM and bone parameters following FMT

**Figure 6:**
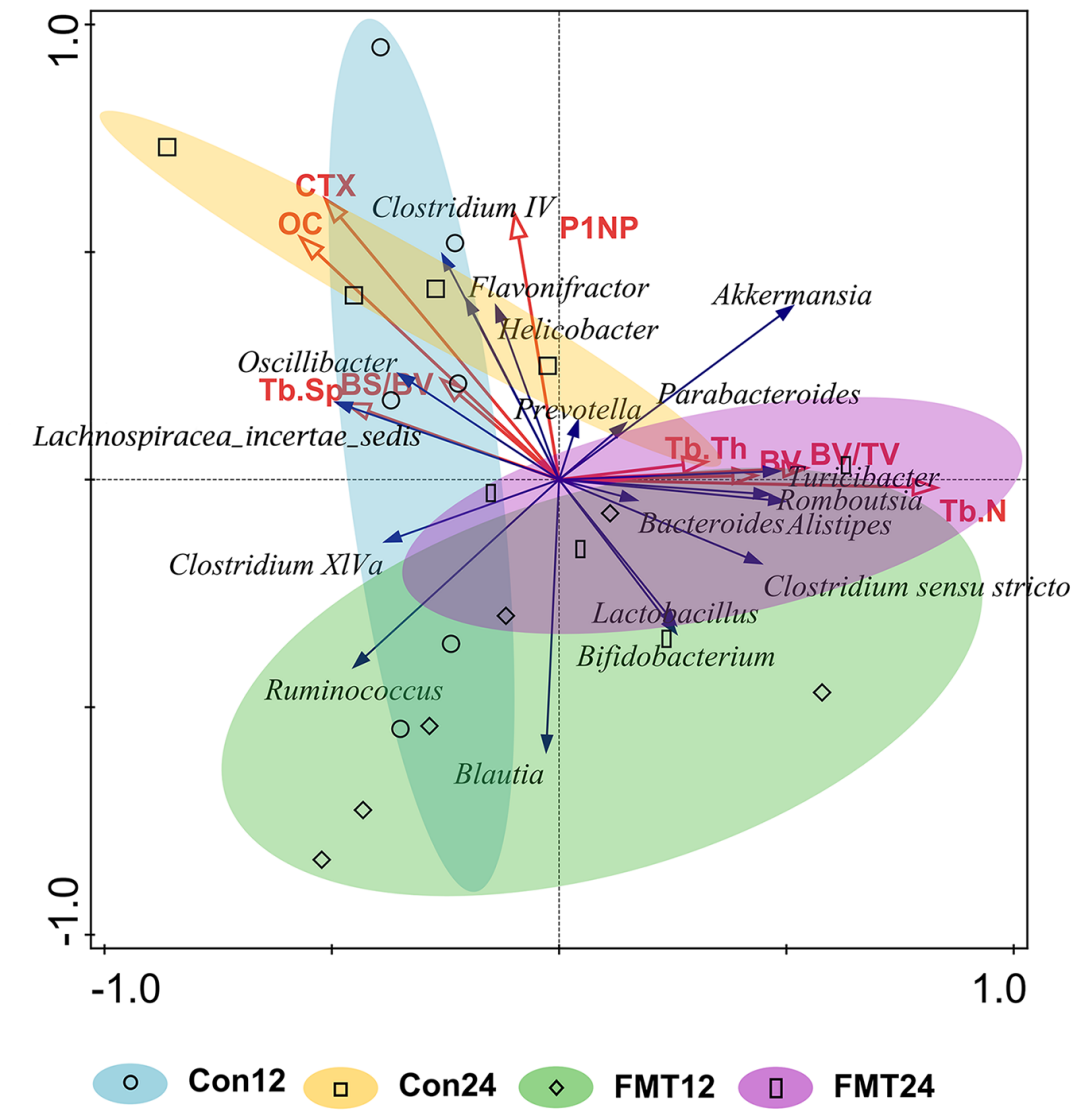
Redundancy (RDA) analysis of gut microbiota and bone parameters following FMT. The blue arrows meant gut microbiota in genus level and the red ones meant bone turnover and histomorphometric parameters. The smaller angle between two arrows means the higher degree of positive correlation. The gut microbiota of the control group and the FMT group entirely separated at 24 weeks. FMT: fecal microbiota transplantation; Con: control; OC: osteocalcin. OC (pseudo-F = 2.5, *P* = 0.022); P1NP (pseudo-F = 1.5, *P* = 0.198); CTX (pseudo-F = 2.7 *P* = 0.012); BV (pseudo-F = 1 *P* = 0.404); BV/TV (pseudo-F = 1.7 *P* = 0.138); BS/BV (pseudo-F = 0.7 *P* = 0.626); Tb.N (pseudo-F = 3 *P* = 0.01); Tb.Th (pseudo-F = 0.8 *P* = 0.552) Tb.Sp (pseudo-F = 1.1 *P* = 0.344)

Redundancy analysis was performed to demonstrate the correlation between GM and bone parameters at 12 weeks and 24 weeks after colonization (Fig. 6). *Prevotella*, *Bacteroides*, *Clostridium *sensu* stricto*, and *Lactobacillus* were positively correlated with Tb.Th, BV, BV/TV, and Tb.N. However, *Helicobacter*, *Oscillibacter*, and *Lachnospiracea_incertae_sedis* were negatively correlated with these parameters. Moreover, GM at the genus level in the control group and the FMT group were partially overlapped at the 12th week, while completely separated at the 24th week.

### GM from young rats modulated the function of GM in aged rats

**Figure 7:**
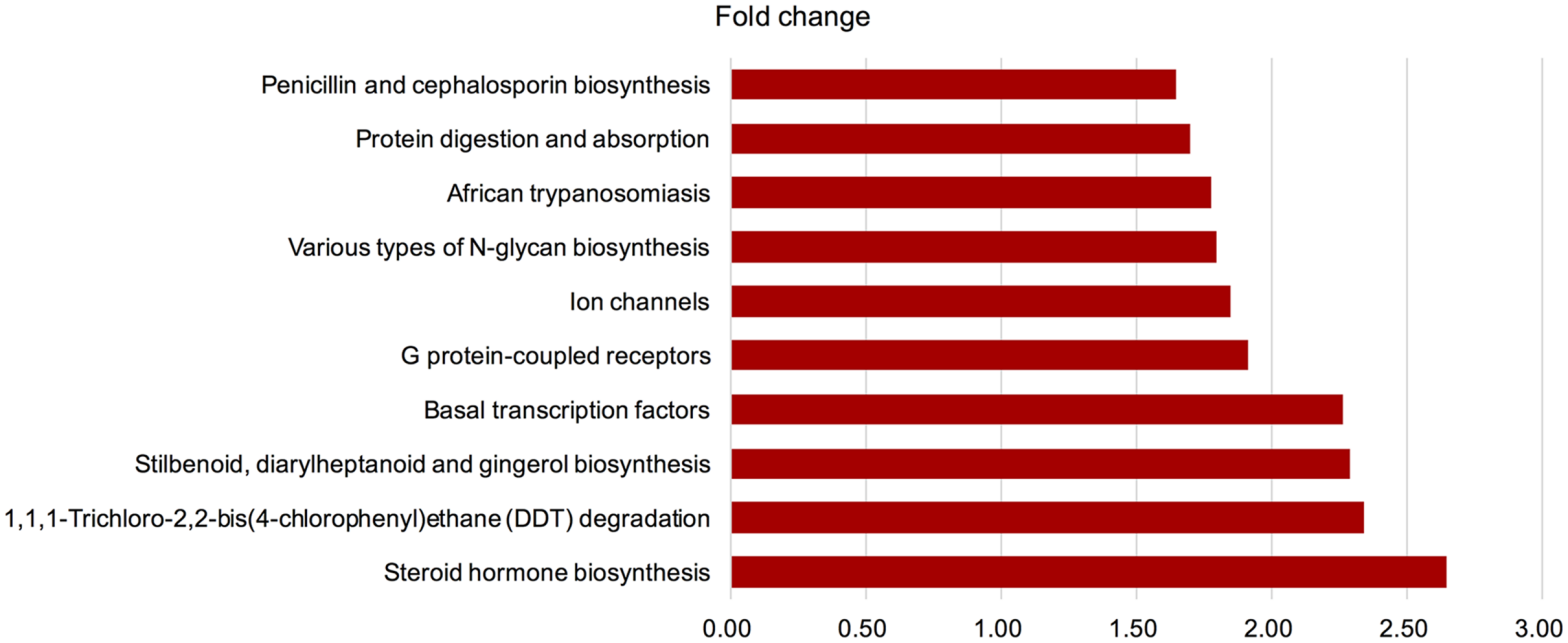
GM from young rats modulated the function of gut microbiota in aged rats. To investigate the function of gut microbiota following FMT, we predicted the pathway based on KEGG database. Significantly different enriched pathways were showed (*P* < 0.05, fold change >1.2 or <−1.2). Four pathways with fold change >2 were found after FMT. FMT: fecal microbiota transplantation.

To investigate the function of GM following FMT, we predicted pathways based on the KEGG database. Significantly differentially expressed genes were found (Fig. 7) (*P* < 0.05, fold change >1.2 or <−1.2). Four pathways with fold change >2 after FMT were identified. Steroid hormone biosynthesis, 1, 1, 1-Trichloro-2, 2-bis(4-chlorophenyl) ethane (DDT) degradation, stilbenoid, diarylheptanoid and gingerol biosynthesis, and basal transcription factors were up-regulated in the FMT group.

### Correlation network analysis between the FMT group and the control group

**Figure 8:**
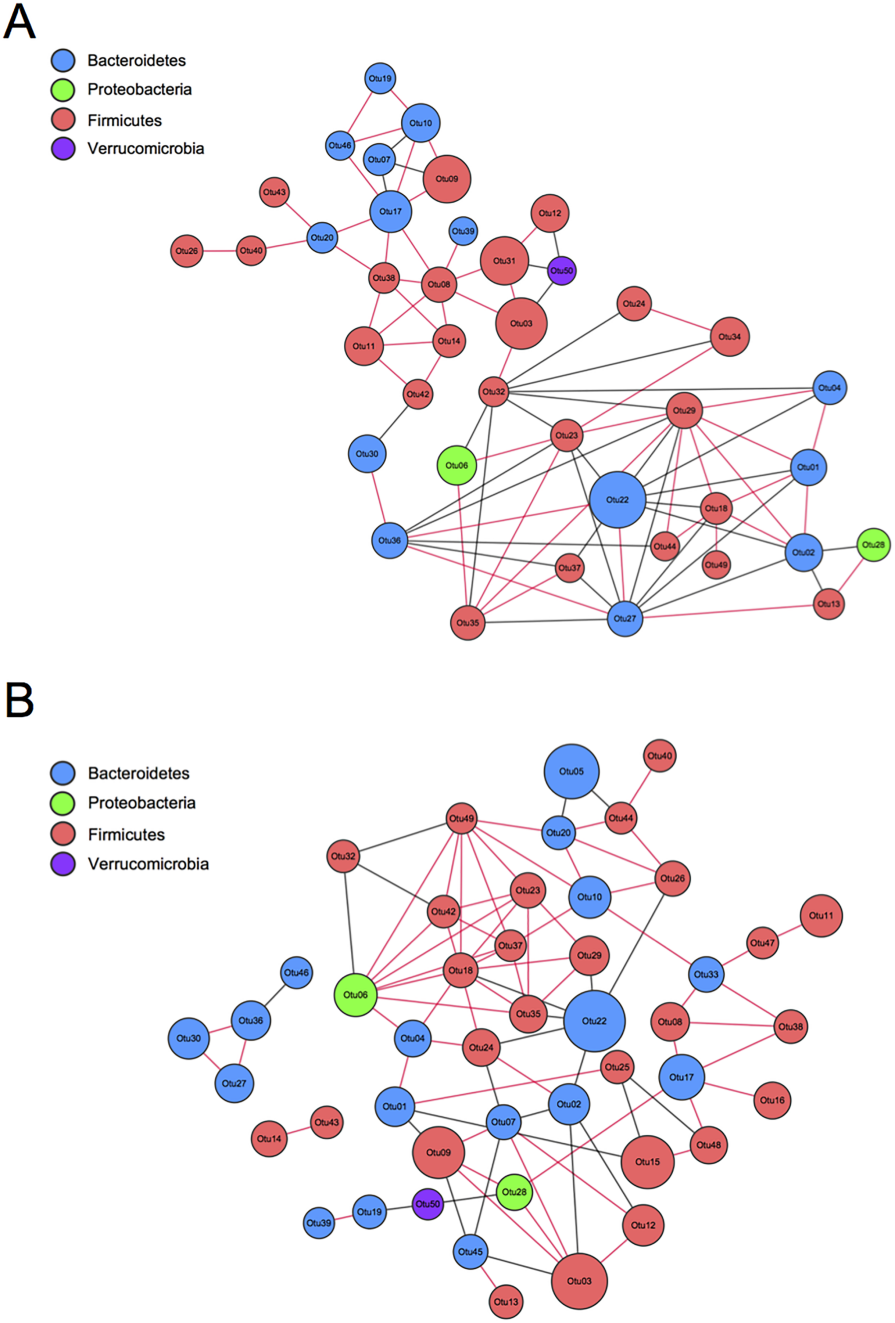
Correlation network analysis of the 50 most abundant OTUs. (A) Twelve weeks following FMT. (B) Twenty-four weeks following FMT. Networks showed significant positive (red) and negative (grey) pairwise correlations between OTUs. OTUs were colored by phylum annotation and sized by mean relative abundances. FMT: fecal microbiota transplantation; Con: control.

To evaluate the correlation between FMT and GM community structure in the recipients, we performed a correlation network analysis of 50 OTUs with the highest abundance to determine the species with key changes. Few correlations and little transitivity between the two groups at the 12th week (Fig. 8A). With the same standard coefficient, a more complex correlation network was constructed 24 weeks after transplantation. A significant correlation and better clustered OTUs were found. OTU06 and OTU18 were selected as the key gene information at the 24th week based on topological properties, which could be annotated to Proteobacteria and Firmicutes, respectively (Fig. 8B). Changes of Firmicutes contributed the most to GM alterations. Proteobacteria had a positive correlation with Firmicutes, while Bacteroidetes had a negative correlation with Firmicutes.

### GM from young rats improved the intestinal structure and barrier function in aged rats

**Figure 9:**
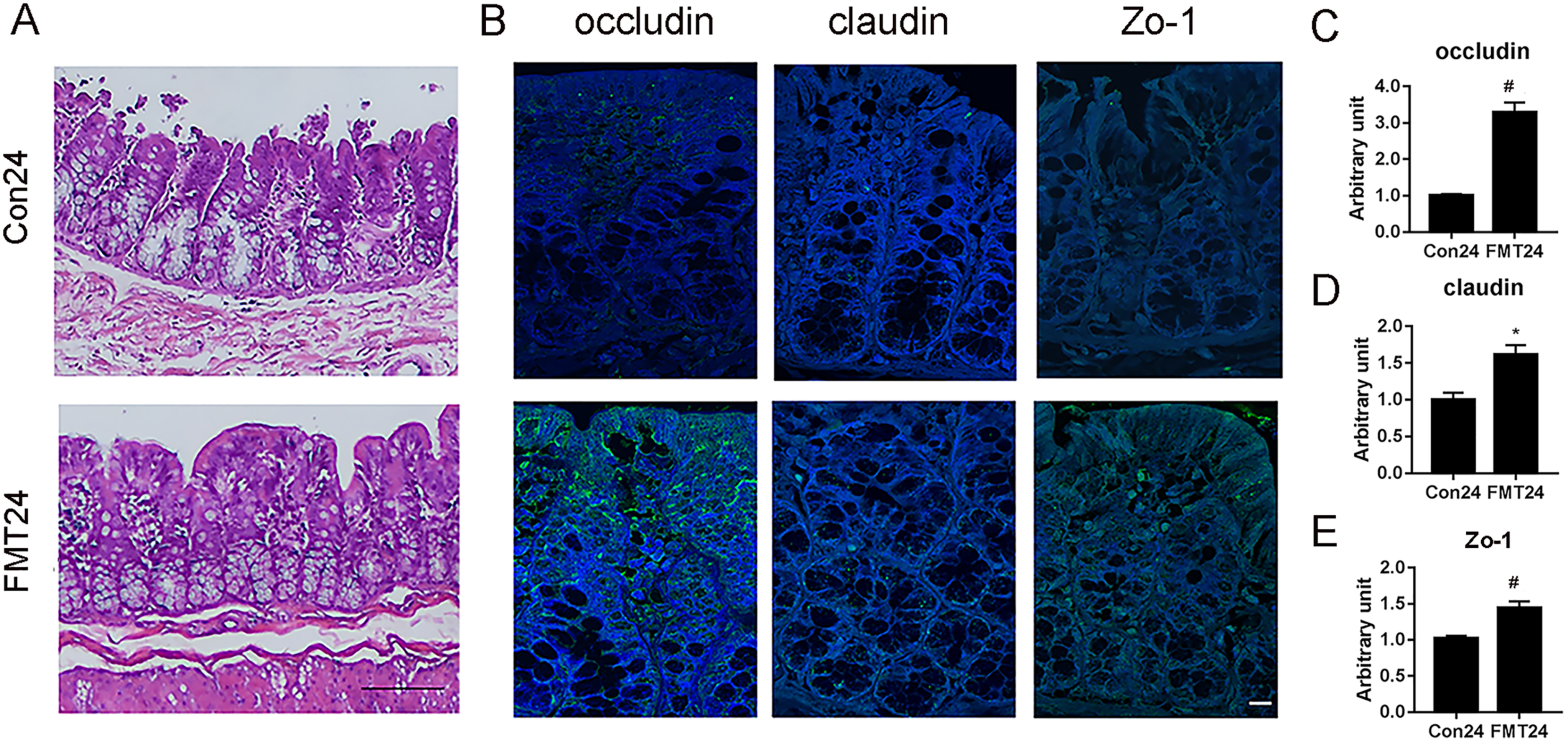
GM from young rats improved the intestinal structure and barrier function in aged rats. (A) HE staining showed the FMT group had less swelling mucosa, sub-epithelial space expansion, and well-arranged villi structure compared with the control at 24 weeks. (B) The expression of occludin, claudin, and ZO-1 increased significantly at 24 weeks following FMT. (C) Occludin was significantly upregulated at 24 weeks following FMT. (D) Claudin was significantly upregulated at 24 weeks following FMT. (E) Zo-1 was significantly upregulated at 24 weeks following FMT. *P < 0.05; ^#^*P* < 0.01 FMT: fecal microbiota transplantation; Con: control.

Intestinal structure and barrier function were proved to be associated with osteoporosis (Schepper et al., 2020). We have manifested that FMT was beneficial for osteoporosis by restoring GM homeostasis. Next, we investigated the intestinal structure and barrier function following FMT. HE staining showed that the FMT group had less swelling mucosa, sub-epithelial space expansion, and well-arranged villi structure at the 24th week (Fig. 9A). Immunohistological staining showed the upregulation of occludin, claudin, and ZO-1 in the FMT group at the 24th week (Fig. 9B). Furthermore, the quantitative results verified that the expression of occludin (*P* < 0.01), claudin (*P* < 0.05), and ZO-1 (*P* < 0.01) significantly increased at 24 weeks following FMT (Figs. 9C–9E). These results suggested that FMT improved the intestinal structure and barrier function in aged rats.

## Discussion

To the best of our knowledge, this is the first study to manifest the feasibility of treating osteoporosis by FMT in aged rats. Our data suggest that FMT could reverse the high bone turnover of senile osteoporosis and alleviate the aging-related bone loss. Moreover, the beneficial effect of FMT on osteoporosis is associated with the improved GM composition and intestinal structure as well as the enhanced barrier function. Therefore, FMT would be developed as a novel and promising intervention against osteoporosis.

FMT seems to be a safe treatment that has manifested good clinical outcomes in several metabolic disorders, although data of long-term FMT are still lacking. (Wang et al., 2018; Tian et al., 2019; Nusbaum et al., 2018; Ohara, 2019; Wang et al., 2020; Paramsothy et al., 2019). In general, the dose and duration of FMT depend on the clinical course and the patient’s comorbidities. Few serious adverse events related to short-term FMT in patients with CDI were reported in clinical trials. Adverse effects occur more frequently in patients with inflammatory bowel disease treated with FMT; Fever, increased C-reactive protein, and bacteremia have been also reported (Ooijevaar et al., 2019). In this study, the aged rats received 24-week FMT from young healthy congeneric rats with a frequency of three times per week. Our results manifested the optimistic feasibilities of FMT in treating osteoporosis. According to previous studies, the duration of FMT is associated with the outcomes. One or two doses of FMT were effective for CDI, and serial FMT was efficacious in inflammatory bowel disease (IBD) (van Nood et al., 2013). In the current study, 12 weeks of FMT was effective on osteoporosis, which was shown by the reduced serum bone turnover markers, while the bone mass difference was detected after 24 weeks of FMT. We speculated that bone turnover markers were more sensitive, which were significantly changed prior to bone mass or GM alteration at the 12th week.

In this study, we found that GM tended to be similar between the aged rats following FMT and the donor rats. Besides, the coprophagic nature of rats might accelerate GM alteration of recipient rats and reinforce the FMT phenotype. Similar results were also found in FMT-treated rats with pulmonary obstructive disease (Liu et al., 2017). However, genus-level differences following FMT were found with increased *Blautia* and decreased *Helicobacter* and *Prevotella*. Blautia is a short-chain-fatty-acids-producing bacterium, which is beneficial for bone health (Zhang et al., 2020). On the contrary, *Prevotella* is associated with inflammatory bone loss (Choi et al., 2016). Previously, we found that *Prevotella* increased rapidly and maintained at a high abundance in the gut of rats with osteoporosis (Ma et al., 2020a). The decreased *Prevotella* by FMT in the current study was consistent with our previous findings.

Correlation network analysis can reduce the complexity of multidimensional data while preserve the required information for interpretation (Kelder et al., 2014). It is used to visualize disease-related perturbations of molecular interactions to provide insights into the key mechanisms underlying disease development. In this study, we found that the correlation network of GM in rats was more complex, and the degree of OTU aggregation was higher at the 24th week compared with the 12th week, which indirectly proved that FMT altered the structure of GM and therefore, possibly induced osteoporosis alleviation. Firmicutes positively related the most with the beneficial effect of FMT on osteoporosis, while Bacteroidetes was negatively related. These findings were consistent with the results from taxa analysis.

Normal intestinal function is of great importance for maintaining body health. The expression of occludin, claudin, and ZO-1 was decreased in osteoporosis (Ma et al., 2020a). A previous study reported that sex steroid depletion decreased bone mass and increased gut permeability, which suggested that osteoporosis might be associated with intestinal barrier function (Li et al., 2016). Glucocorticoids were also found to affect bone mass and intestinal barrier function of mice. Meanwhile, MDY (high-molecular-weight polymer) supplementation, which could protect the intestinal barrier function, alleviated glucocorticoid-induced bone loss, suggesting that the intestinal barrier might be a therapeutic target of osteoporosis (Schepper et al., 2020). Consistent with these studies, our results showed that gut barrier function was crucial to the treatment of osteoporosis. Furthermore, we concluded from the results that FMT improved the histological structure and the tight junction of intestinal epithelium in aged rats, which might play an important role in counteracting osteoporosis by FMT.

Steroid hormone biosynthesis was significantly enhanced following FMT in this study. The gonadal steroids could alter the GM composition, which in turn, mediated the release of estrogen through a steroid hormone biosynthesis pathway (Tetel et al., 2018). Estrobolome, as a gene of GM products, could metabolize estrogen (Plottel & Blaser, 2011), which is enriched in microbial hydroxysteroid deconjugative activity (Kwa et al., 2016). We speculated that the increased steroid hormone biosynthesis following FMT increased estrogen, which might be beneficial for alleviating osteoporosis. In addition, the up-regulation of N-glycan biosynthesis in the intestinal tract was related to Bacteroides and its metabolites in the gut. N-glycan is the nutrient source of immunoglobulin such as IgA (Briliūtė et al., 2019). The increased N-glycan biosynthesis detected in this study might be attributed to the restored GM microenvironment following FMT.

Although the present study assessed of a new, long-duration scheme of FMT therapy for osteoporosis, it has some limitations. First, bacterial load was not examined by qPCR, which might be necessary for revealing the actual changes of the bacteria. Second, key strains were not be screened from the key differential strains for identifying the therapeutic bacteria. Third, GM metabolites were not examined by metabolomics and other technologies, which might be informative to provide more information on the mechanisms of the gut-bone axis.

In summary, GM transplanted from young rats alleviated bone loss in senile osteoporotic rats by improving gut microbiome composition. Furthermore, restoring intestinal barrier function might be an underlying mechanism to alleviate osteoporosis. Our study, for the first time, demonstrates the probability and feasibility of treating osteoporosis by FMT from young rats to aged rats. These data might provide a scientific basis for future clinical treatment of osteoporosis through FMT.

## Supplemental Information

10.7717/peerj.12293/supp-1 Supplemental Information 1 Raw data for Figs. 1 and 2.

10.7717/peerj.12293/supp-2 Supplemental Information 2 Raw data of 16S rRNA sequencing applied for data analyses and were deposited in the National Omics Data Encyclopedia under OEX012577.

10.7717/peerj.12293/supp-3 Supplemental Information 3 Raw data of reads numbers, alpha diversity, information of taxonomy at different levels and difference analysis.

10.7717/peerj.12293/supp-4 Supplemental Information 4 Reads numbers in each sample that enriched in different functional pathway.

10.7717/peerj.12293/supp-5 Supplemental Information 5 Commands and parameters used for Mothur.

10.7717/peerj.12293/supp-6 Supplemental Information 6 Author Checklist.

## Additional Information and Declarations

### DNA Deposition

The following information was supplied regarding the deposition of DNA sequences: The raw data of 16s rDNA sequencing are available in the National Omics Data Encyclopedia (NODE, https://www.biosino.org/node/index): OEX012577.

### Data Availability

The following information was supplied regarding data availability: The raw data are available in the Supplemental Files. The raw data is showing all gut microbiomes in different levels in the control group (OPC) and FMT group (OP).
