# Supplementary material for: Fecal microbiota transplantation mitigates bone loss by improving gut microbiome composition and gut barrier function in aged rats
Source: PeerJ. 2021 Oct 21;9:e12293. doi: 10.7717/peerj.12293 (PMC8542369; doi:10.7717/peerj.12293)
Supplement: Supplemental Information 5 [file peerj-09-12293-s005.docx]

**Commands and parameters used for Mothur**

mothur > make.file(inputdir=., type=fastq, prefix=stability)

mothur > make.contigs(file=stability.files, processors=8)

mothur > screen.seqs(fasta=stability.trim.contigs.fasta, group=stability.contigs.groups, maxambig=0, maxlength=550,minlength=250)

mothur > summary.seqs()

mothur > unique.seqs(fasta=stability.trim.contigs.good.fasta)

mothur > count.seqs(name=stability.trim.contigs.good.names, group=stability.contigs.good.groups)

mothur > pcr.seqs(fasta=silva.bacteria.fasta, start=6000, end=24000, keepdots=F, processors=8)

mothur > rename.file(input=silva.bacteria.pcr.fasta, new=silva.v4.fasta)

mothur > align.seqs(fasta=stability.trim.contigs.good.unique.fasta, reference=silva.v4.fasta)

mothur > screen.seqs(fasta=stability.trim.contigs.good.unique.align, count=stability.trim.contigs.good.count_table, summary=stability.trim.contigs.good.unique.summary, start=428, end=17446, maxhomop=8)

mothur > filter.seqs(fasta=stability.trim.contigs.good.unique.good.align, vertical=T, trump=.)

mothur > unique.seqs(fasta=stability.trim.contigs.good.unique.good.filter.fasta, count=stability.trim.contigs.good.good.count_table)

mothur > pre.cluster(fasta=stability.trim.contigs.good.unique.good.filter.unique.fasta, count=stability.trim.contigs.good.unique.good.filter.count_table, diffs=2)

mothur > chimera.vsearch(fasta=stability.trim.contigs.good.unique.good.filter.unique.precluster.fasta, count=stability.trim.contigs.good.unique.good.filter.unique.precluster.count_table, dereplicate=t)

mothur > remove.seqs(fasta=stability.trim.contigs.good.unique.good.filter.unique.precluster.fasta, accnos=stability.trim.contigs.good.unique.good.filter.unique.precluster.denovo.vsearch.accnos)

mothur > classify.seqs(fasta=stability.trim.contigs.good.unique.good.filter.unique.precluster.pick.fasta, count=stability.trim.contigs.good.unique.good.filter.unique.precluster.denovo.vsearch.pick.count_table, reference=trainset9_032012.pds.fasta, taxonomy=trainset9_032012.pds.tax, cutoff=80)

mothur > remove.lineage(fasta=stability.trim.contigs.good.unique.good.filter.unique.precluster.pick.fasta, count=stability.trim.contigs.good.unique.good.filter.unique.precluster.denovo.vsearch.pick.count_table, taxonomy=stability.trim.contigs.good.unique.good.filter.unique.precluster.pick.pds.wang.taxonomy, taxon=Chloroplast-Mitochondria-unknown-Archaea-Eukaryota)

mothur > dist.seqs(fasta=stability.trim.contigs.good.unique.good.filter.unique.precluster.pick.pick.pick.fasta, cutoff=0.03)

mothur > cluster(column=stability.trim.contigs.good.unique.good.filter.unique.precluster.pick.pick.pick.dist, count=stability.trim.contigs.good.unique.good.filter.unique.precluster.denovo.vsearch.pick.pick.pick.count_table)

mothur > make.(list=stability.trim.contigs.good.unique.good.filter.unique.precluster.pick.pick.pick.opti_mcc.list, count=stability.trim.contigs.good.unique.good.filter.unique.precluster.denovo.vsearch.pick.pick.pick.count_table, label=0.03)

mothur > rarefaction.single(shared=stability.trim.contigs.good.unique.good.filter.unique.precluster.pick.pick.pick.opti_mcc.shared)

mothur > remove.groups(count=stability.trim.contigs.good.unique.good.filter.unique.precluster.denovo.vsearch.pick.pick.count_table, fasta=stability.trim.contigs.good.unique.good.filter.unique.precluster.pick.pick.fasta, taxonomy=stability.trim.contigs.good.unique.good.filter.unique.precluster.pick.pds.wang.pick.taxonomy, groups=Mock)

mothur > dist.seqs(fasta=stability.trim.contigs.good.unique.good.filter.unique.precluster.pick.pick.pick.fasta, cutoff=0.03)

mothur > cluster(column=stability.trim.contigs.good.unique.good.filter.unique.precluster.pick.pick.pick.dist, count=stability.trim.contigs.good.unique.good.filter.unique.precluster.denovo.vsearch.pick.pick.pick.count_table)

mothur > make.shared(list=stability.trim.contigs.good.unique.good.filter.unique.precluster.pick.pick.pick.opti_mcc.list, count=stability.trim.contigs.good.unique.good.filter.unique.precluster.denovo.vsearch.pick.pick.pick.count_table, label=0.03)

mothur > classify.otu(list=stability.trim.contigs.good.unique.good.filter.unique.precluster.pick.pick.pick.opti_mcc.list, count=stability.trim.contigs.good.unique.good.filter.unique.precluster.denovo.vsearch.pick.pick.pick.count_table, taxonomy=stability.trim.contigs.good.unique.good.filter.unique.precluster.pick.pds.wang.pick.pick.taxonomy, label=0.03)

mothur > rename.file(taxonomy=stability.trim.contigs.good.unique.good.filter.unique.precluster.pick.pick.pick.opti_mcc.0.03.cons.taxonomy, shared=stability.trim.contigs.good.unique.good.filter.unique.precluster.pick.pick.pick.opti_mcc.shared)

mothur > rarefaction.single(shared=stability.opti_mcc.shared, calc=sobs, freq=100)

mothur > dist.shared(shared=stability.opti_mcc.shared, calc=thetayc-jclass, subsample=t)

mothur > pcoa(phylip=stability.opti_mcc.thetayc.0.03.lt.ave.dist)
